# A hybrid simulation model of HIV program interventions: from transmission behavior to macroeconomic impacts

**DOI:** 10.1177/20420986251367510

**Published:** 2025-08-20

**Authors:** William Crown, Erin Britton, Moaven Razavi, Yiqun Luan, Senthil Veerunaidu, Jennifer Kates, Gary Gaumer, Monica Jordan, Clare L. Hurley, Allyala K. Nandakumar

**Affiliations:** Center for Innovation in Social Work and Health, School of Social Work, Crosstown Center, 3rd Floor 801 Massachusetts Avenue, Boston University, Boston, MA 02118, USA; Department of Health Policy, School of Population Health, Virginia Commonwealth University, Richmond, VA, USA; Center for Innovation in Social Work and Health, School of Social Work, Boston University, Boston, MA, USA; The Heller School for Social Policy and Management, Brandeis University, Waltham, MA, USA; The Heller School for Social Policy and Management, Brandeis University, Waltham, MA, USA; KFF, Washington, DC, USA; Center for Innovation in Social Work and Health, School of Social Work, Boston University, Boston, MA, USA; Center for Innovation in Social Work and Health, School of Social Work, Boston University, Boston, MA, USA; The Heller School for Social Policy and Management, Brandeis University, Waltham, MA, USA; Center for Innovation in Social Work and Health, School of Social Work, Boston University, Boston, MA, USA

**Keywords:** agent-based modeling, HIV outcomes, hybrid simulation models, macroeconomic outcomes, peer navigators, PEPFAR

## Abstract

**Background:**

Considerable progress has been made in the estimation of drug safety treatment effects—particularly with observational medical claims and electronic medical record data. The use of the Target Trial framework, along with developments in statistical methodology such as doubly robust and G-estimation methods, has improved the ability to draw reliable causal inferences about drug and vaccine safety from observational data. However, such models rarely relate drug safety outcomes to other domains such as economic impacts. As shown by the COVID-19 pandemic, this is a significant limitation with potentially serious and long-term consequences.

**Objective:**

The goal of this paper is to demonstrate that the availability of simulation tools would enable policy-makers to assess drug safety and effectiveness outcomes associated with alternative policies, as well as examine these effects in the context of other domains, such as economic outcomes.

**Design:**

We develop an agent-based simulation model (ABM) of a peer navigator program to support engagement in HIV therapy in Tanzania. Results from the ABM are weighted to reflect the Tanzanian population and fed into the SPECTRUM model. This generates detailed demographic forecasts that are translated into macroeconomic impacts using labor force participation rates from the International Labor Organization, along with an econometric model of gross domestic product.

**Results:**

The ABM simulation estimated that the peer navigation program increased ART participation for men and women by about 12%–15% with no strong trend over time. The impact on VLS, however, was cumulative and significant for both men and women. By year 3, VLS was improved by 33.9 percentage points for women and 32.6 percentage points for men. However, the overall impact of these estimates on mortality was modest—ranging from less than 500 lives per year at the start of the forecast period to about 2500 lives per year in 2030. Consequently, the associated macroeconomic impacts were also small. The relatively modest impacts were due to the limited opportunity for HIV control in Tanzania, which had already met its 95/95/95 goals.

**Conclusion:**

Although the simulated macroeconomic effects of the peer navigator program in Tanzania were modest, the paper demonstrates the feasibility of linking behavioral ABM simulations of program impacts to subsequent demographic effects and, finally, macroeconomic performance. Moreover, given the clinical response in ART and VLS in the exposed population in Tanzania, it is likely that the same peer navigator intervention conducted in another country with a larger at-risk HIV population would be much larger.

## Plain language summary

Simulating the effects of a peer navigation program on antiretroviral treatment and outcomes in Tanzania

Pharmacoepidemiology and drug utilization studies using data from medical claims or electronic medical records are important tools for understanding the performance of drugs and other interventions in real world practice. Typically, however, such studies focus on treated patients and do not address the behavioral aspects of how patients decide upon treatment in the first place.

We use an agent-based simulation model to estimate the impact of a peer navigation program in Tanzania on antiretroviral therapy (ART) participation and adherence, as well as viral load suppression (VLS) for HIV patients. We then estimate the impacts of the program on Tanzanian mortality rates by weighting the patient level estimates to reflect population estimates. Finally, we evaluate the impacts of estimated mortality changes on the productive capacity of the labor force and economic growth. The agent-based simulation finds that the peer navigation program had substantial positive impacts on ART participation for men and women, as well as VLS. However, the ultimate impacts of these effects on mortality and associated economic outcomes were relatively small. This appeared to be due to the relatively small size of the at-risk population who could potentially benefit from the intervention.

To our knowledge, the current study represents the first successful linkage of a behavioral agent-based simulation model of a behavioral health policy intervention with its demographic implications and subsequent macroeconomic ramifications. Although the simulated effects of our study focused on a peer navigator program, rather than a drug per se, the outcomes measured utilization of ART and VLS. The ability to incorporate behavioral aspects of drug treatment patterns through agent-based simulation offers the potential to better understand drug treatment safety and effectiveness, as well as how such outcomes might be improved with augmentative approaches such as peer navigation programs.

## Introduction

Considerable progress has been made in the estimation of drug safety treatment effects—particularly with observational medical claims and electronic medical record data. The use of the Target Trial framework,^1,2^ along with developments in statistical methodology such as doubly robust and G-estimation methods,^3,4^ has improved the ability to draw reliable causal inferences about drug and vaccine safety from observational data. The progress made in estimating drug safety treatment effects is admirable, but there is also an opportunity to relate these estimates to other domains, such as economic outcomes, that have been underutilized.

Moreover, most drug safety studies focus on treated patients and do not address how patients get into treatment in the first place (other than estimation of propensity scores for balancing cohorts). From a public health perspective, treatment effectiveness and safety are necessary but insufficient measures; measures of treatment access and utilization are also fundamentally important for estimating the impact of a therapeutic intervention, because some interventions can increase exposure to a drug or vaccine. Perhaps the most straightforward examples of this are public education campaigns to increase drug or vaccine treatment in the face of disease epidemics and combat public misconceptions about the safety of particular drugs or vaccines. COVID-19 provided an example where vaccination rates were undermined by public misinformation about vaccine safety. The same is true with the re-emergence of measles in the United States. Public education campaigns can help counter such misinformation and increase exposure to safe and effective drug and vaccine interventions.

The COVID-19 pandemic also provides a recent important example of when it was necessary to evaluate the safety and effectiveness of vaccines in the context of other interventions, such as social distancing and masking. Simulation is a useful methodology for problems characterized by nonlinearities, feedback loops, and emergent behavior.^5^ Epidemics have all of these characteristics. Yet, very few countries had established a simulation infrastructure that would enable policy-makers to evaluate different combinations of interventions without actually implementing them and observing the consequences. We learned in retrospect that vaccination, coupled with masking and a focus on key populations at risk, was highly effective and safe.^6–9^ However, the widespread use of school closures and online instruction has had long-term effects on the learning of children.^10^ Disruptions to the workplace also had enormous negative economic impacts.^11^

### Economic implications of health interventions

A large literature has demonstrated that investments in human capital and health are associated with economic growth.^12–18^ For example, Rocco et al. find that each 10 percentage point increase in mortality rates is associated with an 8–13 percentage point decline in gross domestic product (GDP).^19^ This implies that vaccines and drug interventions with substantial impacts on mortality reduction would be expected to have large positive impacts on economic growth. However, results from epidemiologic studies of drug safety and effectiveness are rarely linked to broader economic considerations. Likewise, in the economics literature on the economic and educational impacts of health investments, it is rare to study the economic impacts of specific interventions—whether they be programs or medical interventions such as drugs or vaccines.

PEPFAR, the US global AIDS program, is the largest public health initiative ever dedicated to combating a single disease^20–23^ and has been credited with saving more than 25 million lives.^24^ PEPFAR does many things, including training healthcare workers and strengthening health systems in recipient countries. Perhaps most importantly, it has provided antiretroviral therapy (ART) and prevention services to millions of people. Many studies have confirmed the life-saving effects of PEPFAR activities.^25–29^

In a series of recent studies, Gaumer et al. found that PEPFAR investments were associated with large, significant reductions in all-cause mortality.^30^ In a related study, Gaumer et al. found that PEPFAR was associated with reductions in maternal and child mortality and increases in childhood immunizations.^31^ Gaumer et al. found that PEPFAR resulted in significant, positive impacts on educational levels and GDP.^31^

In the current study, we sought to understand if PEPFAR’s impacts could be enhanced with a peer navigation program that would support patients getting tested for HIV and taking antiretroviral drugs, and remaining on therapy if they were found to be positive for HIV. Peer navigation programs involve recruiting individuals to influence people within their social networks (family members, friends, colleagues at work and school) to seek testing and comply with treatment. We also sought to demonstrate the feasibility of connecting the individual outcomes resulting from behavioral responses to peer navigators to macroeconomic outcomes such as labor force participation and GDP growth.

### Why are simulation methods needed?

Healthcare delivery systems are complex and often highly fragmented. They are social systems consisting of many different groups or agents, including governments, payers, and providers, responsible for delivering healthcare services to patients.^5^ Social systems are inherently complex because each of the agents in these systems makes decisions, interacts among themselves, and interacts with other parts of the system in an interdependent manner. The complexity of such interactions leads to emergent behavior that is difficult to predict. Although modeling approaches such as decision trees and Markov models have been widely used as methods to evaluate healthcare interventions such as vaccines or drug treatments, these approaches may not be sufficient for analyzing complex healthcare delivery systems. Instead, dynamic simulation modeling offers advantages, given recent advances in accessible computing power and data analytics that make it possible to simulate the impact of system interventions on healthcare delivery systems without costly and time-consuming direct experimentation.

The results of such simulation models can anticipate the comparative effectiveness or safety of a novel system intervention as well as its cost-effectiveness. Three dynamic simulation modeling methods are well suited for, and commonly applied to these types of problems: system dynamics (SD), discrete event simulation, and agent-based simulation model (ABM).^32–35^ For this analysis, we used ABM, as described below.

### Agent-based simulation models

ABM is a simulation method for modeling dynamic, adaptive, and autonomous systems.^5^ At the heart of an ABM model, there are autonomous and interacting agents who can react to the changes in their environment and actively make decisions. In healthcare contexts, the most notable agents are patients, households, and healthcare providers who interact with one another. Each agent exists within an environment, and their next actions are based on the current state of their environment. An agent senses its environment and behaves accordingly, on the basis of defined decision rules. The three core concepts that form the basis for an ABM are agency, dynamics, and structure. Dynamics means that both the agents and their environment can change, develop, or evolve. Structure is emergent from agent interaction. How human populations tend to congregate and interact in certain locations, such as the workplace, marketplace, or school, is an example of structure. Patient-centered ABMs enable researchers to simulate the health-seeking behaviors of the agents in a changing environment, or response to incentives and stimuli, along with impacts they may have on other agents’ behaviors or even on their environment. ABM is a rapidly maturing health modeling technique that is well suited for addressing public health planning and policy needs, as well as supporting healthcare infrastructure investment decisions. The ability of ABMs to simulate the effects of introducing a program and changing its features is a powerful tool for assisting public health planning and program implementation.

### The need for hybrid simulation models

Simultaneous or sequential estimates of the effects of health-seeking behaviors and outcomes on subsequent social and economic indicators require a hybrid modeling approach. Standalone simulation models have been widely used to model the transmission of HIV.^36^ However, the use of ABMs to model the behavioral response to HIV programs has been limited,^37^ and there are no examples of where results from ABMs have been cascaded (used as inputs) to other modeling tools to examine their implications for demographic and macroeconomic impacts. Any large-scale public health investment program, such as PEPFAR, will operate through the response of agents to its initiatives. For example, the establishment of a new HIV clinic will require individuals to travel to the clinic to get tested. Those who travel to the clinic and learn that they are HIV positive must decide whether to initiate ART and continue on ART to maintain their health until they are no longer infectious. Some individuals will discontinue ART and could become sick and/or infectious once again. The point is that much of the impact of public health initiatives is the cumulative result of the behavioral responses that the program triggers in a large number of individuals or agents. Ultimately, the safety and effectiveness of a drug or vaccine are strongly influenced by the behaviors of patients and providers. The ability to model these behavioral responses and understand their impacts is key to understanding the dynamics of how a program or drug can achieve its intended effects. Estimates of treatment effectiveness and safety are critically important in and of themselves. However, using simulation, it is also possible to examine how other factors, such as social stigma, interact with these treatment effectiveness or safety estimates to anticipate the ultimate impact of a drug or vaccine on public health outcomes. Consequently, it is critically important to develop ABM simulation capabilities to assist large-scale public health programs such as PEPFAR in planning and program evaluation.

The ultimate goal of any public health investment is to reduce morbidity and mortality in the target population. Much of the research on HIV interventions has focused on estimating the effects of such interventions on patient outcomes using statistical methods from epidemiology and econometrics, as well as simulation methods. Research on the economic impacts of specific HIV programs, such as PEPFAR, is far more limited. The most recent and ambitious effort to simulate the macroeconomic impacts of HIV investments is the work of The Economist Global Impact Team. Their study links demographic estimates of the impacts of HIV spending using the SPECTRUM model to estimate demographic impacts and subsequent macroeconomic impacts.^38,39^ SPECTRUM is a sophisticated HIV policy analysis model that incorporates the ability to simulate the demographic effects of aggregate inputs such as program spending or changes in ART or viral load suppression (VLS) at a country-specific level.^39^ It incorporates detailed demographic modeling dynamics based upon population structure by age and sex, birth rates, and impacts of ART and VLS on mortality by age and sex. In this analysis, we extend the work of the Economist Impact report^38^ by adding an ABM component to the model. Rather than pre-specifying the levels of the program inputs that feed into SPECTRUM, we use an ABM to simulate them. In so doing, we can trace the behavioral responses to a public health intervention (using ABM) on demographic dynamics (using SPECTRUM). In the final step, we relate these demographic changes to changes in labor supply and GDP. We develop and test this cascaded hybrid modeling capability by simulating a peer navigator program in Tanzania.

## Methods

We developed an ABM to simulate the effects of a peer navigation program on HIV treatment and outcomes, and then aggregate and weight the individual-level results from the ABM to reflect national estimates. These are then subsequently used to estimate the macroeconomic impacts resulting from the peer navigation program. Estimated changes in adult ART retention were fed into the SPECTRUM HIV policy simulation model to project population changes over a 10-year period. In the final step, the projected demographic data were linked with labor force participation rates to generate estimates of labor supply that were fed into an econometric model to estimate the effects on GDP.

### Simulating a peer navigator program in Tanzania

To pilot the feasibility and utility of this hybrid modeling approach, we build on prior research demonstrating the potential for married women with high agency to positively influence their social networks through peer navigation.^40^ Peer navigator programs are those that attempt to intervene with patients through influential people in the patients’ network. The idea is that married women influence the care-seeking behavior of spouses and other family members, increasing the probability that they will get tested for HIV, get on ART, and stay on ART. However, these same women also influence friends and social connections at work, school, and other settings. Moreover, some portion of the patients who are influenced by the peer navigators, in turn, become peer navigators themselves and influence others. The probabilistic nature of these interactions and the complexity of social networks create “emergent behavior,” which is the result of the interactions that are impossible to predict without the use of simulation. The intervention in our ABM was based on recent evidence from a randomized controlled trial in Kenya, which demonstrated the potential of peer navigation to improve care re-engagement and retention. In this trial, Geng et al. evaluated the effects of different strategies to influence treatment initiation and retention and found that peer navigators meaningfully improved treatment success among those with lapses in continuity.^41^

#### Tanzania: An illustration

**Figure 1.**
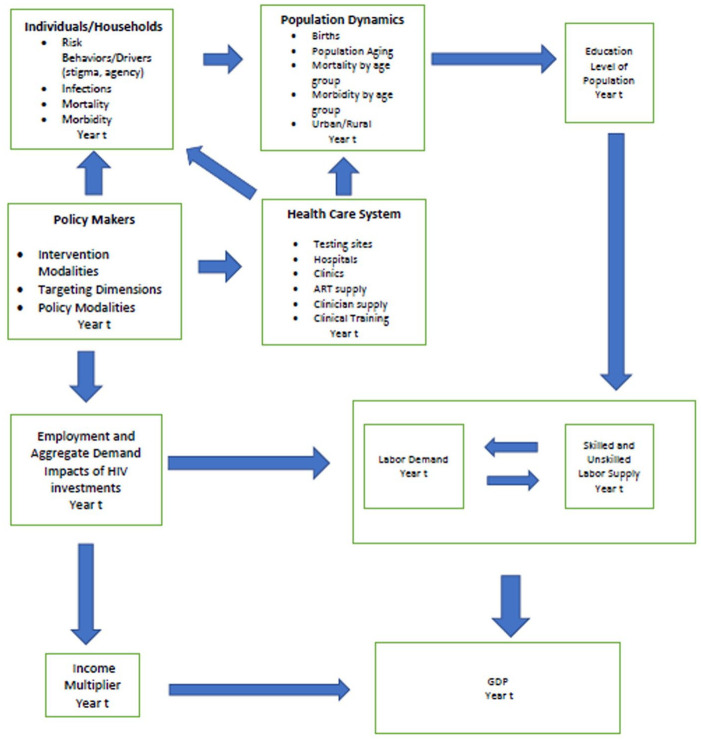
ABM and system dynamics model of HIV policy, HIV mortality, employment, and gross domestic product. ABM, agent-based simulation model.

Given these results, we evaluated the effects of a peer navigator program on demographic and macroeconomic outcomes using a hybrid approach linking an ABM, SPECTRUM, and an econometric model of GDP using data from Tanzania. The structure of the model is illustrated in Figure 1.

First, consider the policy-makers component in Figure 1. Working clockwise around the figure, the model assumes that health investments in HIV programs such as PEPFAR create improvements in healthcare infrastructure, which improves access to care, as well as behavioral incentives for households and individuals. For example, building a new clinic that supports the provision of ART increases the supply of therapy for patients and households in the proximity of the new clinic. An ABM could also model the effects of clinic proximity on patient behavior by assigning probabilities that patients will come into the clinic to be tested, which vary by distance. Thus, the healthcare system and the individual/household components are where the ABM takes place. Ultimately, the actions of agents in these two components result in changes in HIV testing and awareness, treatment with ART, and VLS. In turn, these changes in health processes and outcomes as outputs from the ABM would be expected to impact mortality, an important element of the Population Dynamics component.

Several studies have demonstrated that large public health investments to address HIV in low- and middle-income countries have had positive impacts in reducing mortality.^30^ Mortality reductions directly influence the size of the labor force with subsequent implications for the productive capacity of economies. Despite the size of the positive impacts of public investment in HIV programs on population health, the literature estimating the macroeconomic benefits of such investment is relatively small.^12,15,16,38,42^

In addition to the direct impact of mortality reductions on labor force size and the productive capacity of economies, economic growth, in the long run, also enhances the ability of a society to invest in further educational and healthcare infrastructure, creating a positive feedback loop stemming from the original HIV program investments.^15^ Finally, it is plausible to expect that any large-scale public program to address HIV would have direct and indirect demand-side macroeconomic impacts as spending from such programs works its way through countries’ economies over time. Specifically, investments in healthcare infrastructure generate income for healthcare workers, which is then spent, creating subsequent income for people working in other sectors. This income is then re-spent and, via Keynesian macroeconomic multipliers, generates potential benefits worth multiples of the original expenditure.

#### Integrating ABM with SPECTRUM

Rather than building a Population Dynamics component from scratch, we elected to use the existing capabilities of the SPECTRUM model. The SPECTRUM model was specifically developed to support HIV policy analyses. The Population Dynamics component of SPECTRUM forecasts single-year population levels by sex over a 10-year forecast period using country-specific historical data on population age/sex structure, birth rates, and mortality rates. An HIV disease transmission model relates the transmission of the disease and mortality to VLS. Thus, the outputs from the ABM can be aggregated from the individual agent level and weighted to reflect national effects. These can then be entered as inputs into SPECTRUM to model their effects on the forecasted age/sex structure of the population. To link the ABM results to the SPECTRUM model, the distribution of agents used for the simulation was scaled to the “real-world” population distribution of men and women in Tanzania. The weighted outputs from the ABM were then incorporated into SPECTRUM by modifying the percent of patients living with HIV receiving ART in the adult ART program statistics component of the AIDS Impact Model (AIM). The HIV disease transmission model within SPECTRUM then translated these inputs into the associated demographic changes in the population distributions of men and women by age.

### Linking population dynamics to economic outcomes

**Figure 2.**
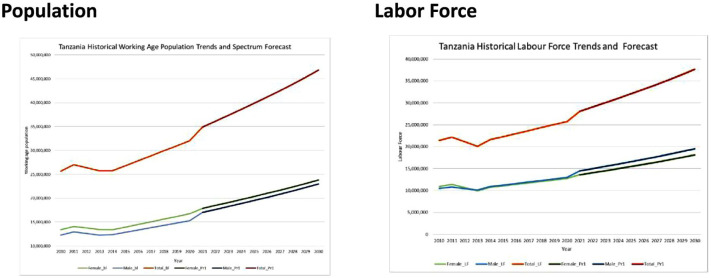
Tanzania’s historical population trends, baseline spectrum forecast, and associated labor force estimates.

Population dynamics, in turn, determine the size of the population moving through the various levels of the educational system and have a strong influence on the formation of human capital that contributes to the productivity of the labor force. Population dynamics also determine the age- and gender-specific size of the labor force. Together, these two components account for the aggregate human capital available in the labor force to produce the goods and services needed by the economy. We apply age/sex labor force participation rates from the International Labor Organization (ILO)^43^ to the SPECTRUM demographic forecasts to generate estimates of the size of the labor force over the forecast period. The historical and forecast values of the population and labor force size for Tanzania are shown in Figure 2. The left panel shows the historical and forecast population values, while the right panel reports the historical labor force values and forecasts after applying the ILO labor force participation rates to the population data.

We then estimate an econometric model of GDP and use it to forecast GDP levels in response to changes in the productive capacity of the labor force, using a similar specification to recent work in the literature.^12,38^

Due to limitations in the number of historical observations available for Tanzania, we estimate the model using observations from 31 PEPFAR countries that prepare Country Operational Plans (COPs), and then evaluate how well the model predicts historical levels for Tanzania’s GDP.^44^ We chose PEPFAR COP countries for the modeling of GDP because these countries often have the greatest unmet HIV needs and generally receive the largest budget allocations. Tanzania is one of the 31 COP countries.

The GDP model, estimated in double-log form as a Cobb-Douglas production function, explains 94% of the variation in the log of GDP over time. GDP is modeled as a function of the total size of the labor force, secondary school enrollment, urban population percentage, and yearly trends. The pattern of predicted and actual values when the historical observations for Tanzania are run through the model to estimate historical levels of GDP is very similar, indicating that the model appears to be well-calibrated for Tanzania, even though it was estimated using data from the broader sample of COP countries. Estimation and calibration details for the GDP model are reported in the Supplemental Materials.

### ABM for modeling peer navigation in Tanzania

The objective of the ABM was to build upon our earlier work on the agency of married women by simulating an intervention that targets married women to influence engagement and retention in HIV care. Agency and knowledge distributions for married women were estimated using Population-Based HIV Impact Assessment (PHIA) surveys in Malawi, Tanzania, and Zambia, 2016–2017.^40^ We used outputs from the ABM as inputs into the SPECTRUM model to estimate population effects in Tanzania.

The ABM intervention modeled the ability of high-agency married women as peer navigators to influence family and community engagement and retention in care. Community components included variation in household size, composition, community density, and social connectedness. Employment and education were hypothesized to drive routine movement to other locations, generating social interactions. In addition, care seeking at clinics generated decision-making related to testing and treatment uptake.

In the model, we simulate health-seeking behavior of the agents as they make decisions dynamically about whether to migrate, seek care, adhere to their treatment plan, or return for follow-up appointments. Individuals in the model are assumed to have specific family and community networks. For example, one individual might live in a household with 3 people and have 14 social connections. Another might live in a household with 5 people and have 7 social connections. Social interactions in various settings, such as school or work, present opportunities to leverage the empowerment of married women to influence their household and social peers. High-agency women engaged in care enter the pool of agents to be selected by the model as peer navigators.

In the scenario used for this research, 2000 households and 10,000 married women agents participated in the ABM simulation. We also tested other model scenarios with smaller agency sizes, which indicated that model results converged with approximately 1000 households and 5000 married women agents. However, we report the results from the simulation with the largest agent sizes of 2000 households and 10,000 married women. The models simulated the impacts of the peer navigator program over 1, 2, and 3-year periods.

## Results

**Figure 3.**
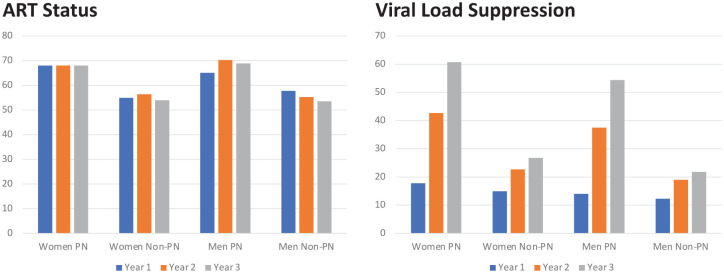
ABM simulation of the peer navigator program. ABM, agent-based simulation model.

As shown in Figure 3, the ABM simulation estimated that the peer navigation program increased ART participation for men and women by about 12%–15% with no strong trend over time. The impact on VLS, however, was cumulative and significant for both men and women. By year 3, VLS was improved by 33.9 percentage points for women and 32.6 percentage points for men.

**Figure 4.**
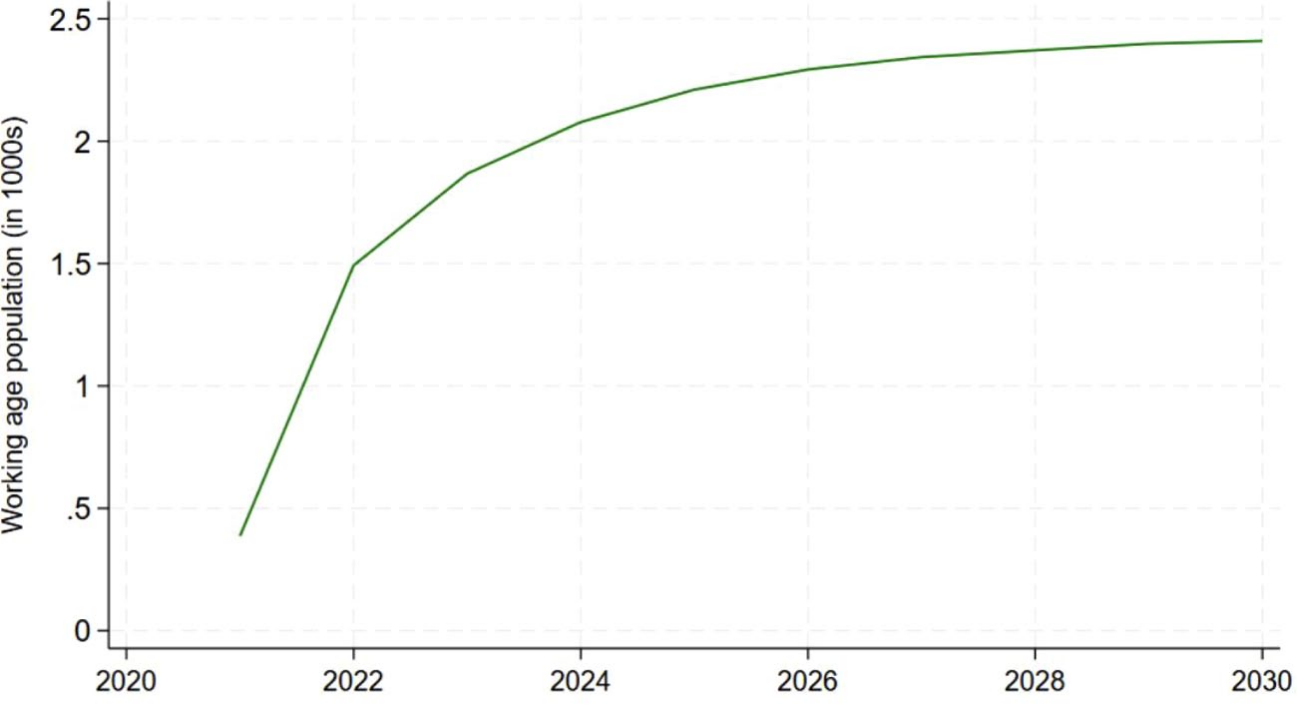
Change in Tanzania working-age population resulting from the peer navigator program, estimated by the SPECTRUM model.

**Figure 5.**
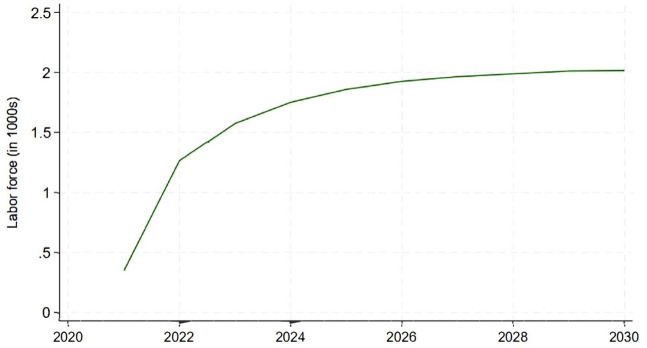
Labor force gains resulting from the peer navigator program.

**Figure 6.**
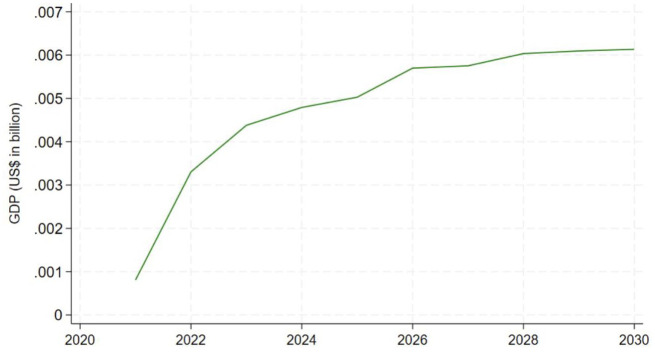
GDP gains resulting from the peer navigator program. GDP, gross domestic product.

The estimates of the effects of the peer navigator program on the population of the age-specific labor force, relative to the baseline produced by SPECTRUM, are shown in Figure 4. In contrast to the large gains in VLS resulting from the peer navigator program, the impacts on overall mortality in Tanzania are modest—ranging from less than 500 lives per year at the start of the forecast period to about 2500 lives per year in 2030. The translation of these population gains into labor force gains using age- and sex-specific labor force participation rates from the ILO is shown in Figure 5. Finally, the impact on Tanzanian GDP is shown in Figure 6. Given the modest impact of the peer navigator program on population gains, the impacts on labor force size and GDP were correspondingly small. Nevertheless, the exercise demonstrates the feasibility of linking a hybrid ABM model that simulates the impact of a policy intervention on individual and household health-seeking behaviors, translating those impacts into demographic changes, and, finally, translating the demographic changes into macroeconomic outputs.

## Discussion

To pilot estimating the macroeconomic impact of public investments in HIV programs, we developed a hybrid simulation model of GDP in Tanzania linking the results of an ABM simulating the change in adult ART retention through a hypothetical peer navigation program with SPECTRUM population projections and labor force participation rates from the ILO. We found that recruiting married women who reach VLS to lead a community-based peer navigation program promoting ART re-engagement increased ART participation and VLS among men and women; however, over a 10-year time horizon, modest mortality reductions had a modest impact on labor force size and GDP. Although the ABM estimated that the program was highly effective in improving ART status and HIV VLS, it applied to a relatively small group of people—those in the networks of high-agency married women. In addition, Tanzania is a country that has already met its 95/95/95 goals. As a result, Tanzania has limited opportunities for further large gains in mortality reduction. The Tanzania results illustrate that macro-level trends may sometimes obscure important gains toward program-specific goals, particularly when interventions target small, vulnerable populations.

Disaggregating the overall trend in Tanzania by age and sex indicates that women outpace men and children by an average of 5 and 25 percentage points across the care cascade, respectively.^38^ While women’s agency and social capital may provide a leverage point to achieve gap closure for men and children, the concept of diminishing marginal returns implies that the opportunities for further gains in ART coverage and VLS are limited in Tanzania. The estimated modest gain in health outcomes results in a limited increase in population size and hence macroeconomic performance. This is because only the relatively small segment of the population that has not already met the 95/95/95 goals can benefit from the incremental interventions. The ABM results clearly show that the peer navigator program resulted in substantial gains in VLS in Tanzania. Likely, a similar intervention carried out in another country whose HIV epidemic is much less well controlled would have resulted in much larger impacts on mortality and subsequent impacts on labor supply and GDP. Consequently, despite the modest impacts on mortality, labor supply, and GDP in Tanzania, leveraging the social capital of women may be an important strategy to achieve HIV care cascade goals for men and children in countries where HIV is less well controlled.

## Limitations

This study has demonstrated the feasibility of linking an ABM policy simulation of an HIV peer navigator program to macroeconomic impacts through the SPECTRUM health and demographic model. However, the macroeconomic model would benefit from further refinements on multiple fronts. The current version of the model assumes that the age- and sex-specific labor force participation rates remain at their 2020 values over the forecast period until 2030. Similarly, trends in urbanization rates and school attendance rates are fixed at their 2020 levels. Given the slow rate of change in socio-demographic variables measured at the population level, and the fact that the forecast period extends only to 2030, we do not anticipate significant changes in the estimated macroeconomic indicators under more realistic scenarios with time-varying rates. Nevertheless, for simulations over a longer time frame, it would be preferable to replace these assumptions with estimates that recognize the endogeneity of labor force participation rates, urbanization, education, and GDP over time. This could be done by estimating an econometric model that incorporates the endogeneity of labor force participation, urbanization, education, and GDP over time. In addition, the current version of the model does not account for the long-term feedback effects of rising GDP on the ability of the economy to invest in its healthcare system. This would be expected to augment the effects of the modeled intervention over time. Feedback effects could be incorporated by replacing the current macroeconomic model with an SD model that more completely reflects the schematic in Figure 1. This would also include the incorporation of demand-side impacts resulting from rising incomes and multiplier effects as a result of health investment and rising educational levels. These effects are likely sizable over longer forecast periods. Due to underestimation of demand-side stimuli, the current conservative GDP projections produced by the model should be considered lower-bound estimates that mainly reflect supply-side impacts of health investment on the productive capacity of economies, and only through increases in labor force participation. In general, simulation models are only as good as the data and assumptions upon which they are constructed. When constructing simulation models, testing their ability to replicate historical data is critical. However, the most important test is to assess their ability to correctly forecast the results of policy interventions as the real-world evidence unfolds.

## Conclusion

To our knowledge, this study represents the first successful linkage of a behavioral ABM to simulate the effects of a health policy intervention on its demographic implications and subsequent macroeconomic ramifications. Although the simulated effects of the peer navigator program were modest, the study demonstrates the feasibility of a general approach for simulating the effects of future policy interventions with potentially larger impacts.

## Supplemental Material

sj-docx-1-taw-10.1177_20420986251367510 – Supplemental material for A hybrid simulation model of HIV program interventions: from transmission behavior to macroeconomic impacts Supplemental material, sj-docx-1-taw-10.1177_20420986251367510 for A hybrid simulation model of HIV program interventions: from transmission behavior to macroeconomic impacts by William Crown, Erin Britton, Moaven Razavi, Yiqun Luan, Senthil Veerunaidu, Jennifer Kates, Gary Gaumer, Monica Jordan, Clare L. Hurley and Allyala K. Nandakumar in Therapeutic Advances in Drug Safety

sj-docx-2-taw-10.1177_20420986251367510 – Supplemental material for A hybrid simulation model of HIV program interventions: from transmission behavior to macroeconomic impacts Supplemental material, sj-docx-2-taw-10.1177_20420986251367510 for A hybrid simulation model of HIV program interventions: from transmission behavior to macroeconomic impacts by William Crown, Erin Britton, Moaven Razavi, Yiqun Luan, Senthil Veerunaidu, Jennifer Kates, Gary Gaumer, Monica Jordan, Clare L. Hurley and Allyala K. Nandakumar in Therapeutic Advances in Drug Safety

## Declarations

**Ethics approval and consent to participate:** Not applicable. This study involved the analysis only of county-level summary data. No ethical determinations were required.

**Consent for publication:** Not applicable. No patient-level data were included in the study.

**Availability of data and materials:** Data on labor force participation rates in Tanzania are available from the International Labor Organization at https://ilostat.ilo.org. Data on Tanzania’s Gross Domestic Product are available from the World Bank at https://datatopics.worldbank.org/world-development-indicators. PHIA data used for the ABM are available from Columbia University at https://phia.icap.columbia.edu. The SPECTRUM model used for demographic forecasts is freely downloadable at https://avenirhealth.org.
