# Supplementary figures and images for: A hybrid simulation model of HIV program interventions: from transmission behavior to macroeconomic impacts
Source: Ther Adv Drug Saf. 2025 Aug 20;16:20420986251367510. doi: 10.1177/20420986251367510 (PMC12368329; doi:10.1177/20420986251367510)

Supplemental Figure 1. GDP Model Results


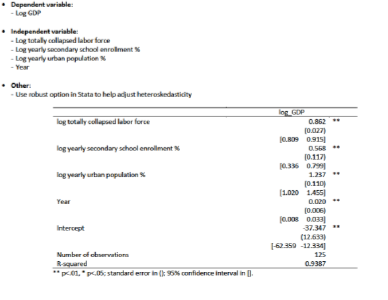

Supplement: sj-docx-1-taw-10.1177_20420986251367510 – Supplemental material for A hybrid simulation model of HIV program interventions: from transmission behavior to macroeconomic impacts [file sj-docx-1-taw-10.1177_20420986251367510.docx]

Supplemental Figure 2. Calibration of GDP Model for Tanzania


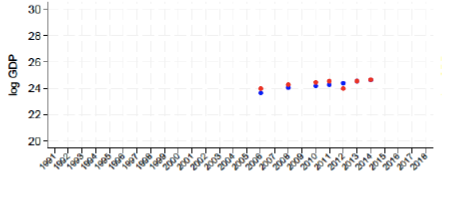

Supplement: sj-docx-2-taw-10.1177_20420986251367510 – Supplemental material for A hybrid simulation model of HIV program interventions: from transmission behavior to macroeconomic impacts [file sj-docx-2-taw-10.1177_20420986251367510.docx]
